# Concomitant Bilateral Inferior Gluteal Lymph Node Involvement in Metastatic Prostate Adenocarcinoma Detected on ^68^ Gallium-Prostate-Specific Membrane Antigen Positron Emission Tomography/Computed Tomography

**DOI:** 10.1055/s-0044-1779281

**Published:** 2024-02-06

**Authors:** Parth Baberwal, Sunita Sonavane, Sandip Basu

**Affiliations:** 1 Radiation Medicine Centre, Bhabha Atomic Research Centre, Tata Memorial Hospital Annexe, Jerbai Wadia Road, Parel, Mumbai, Maharashtra, India; 2 Homi Bhabha National Institute, Mumbai, Maharashtra, India

**Keywords:** metastatic prostate adenocarcinoma, inferior gluteal lymph node, ^68^ Ga-PSMA-11 PET-CT

## Abstract

An unusual and unique case of prostate adenocarcinoma with involvement of bilateral inferior gluteal lymph nodes is reported. The patient was a 42-year-old male, with conventional prostatic adenocarcinoma (Gleason score: 5 + 4 = 9), who, during disease progression with rising serum prostate specific antigen levels following medical androgen deprivation therapy, demonstrated new prostate-specific membrane antigen expressing metastatic intermuscular deposits in the bilateral gluteal region, subsequently proven to be bilateral inferior gluteal nodal metastasis. A therapeutic implication to this may be that these nodes usually fall beyond the range covered by the therapeutic radiation field coverage where external radiotherapy is the advocated modality of choice and are not easily reachable through standard surgical procedures. As a result, they could have an impact on the way patients are clinically treated and on their prognosis.

## Introduction

Prostate cancer is one of the commonly encountered male cancers in the world. 1 The diagnosis is made with the help of transrectal ultrasound (TRUS)-guided biopsy and serum prostate-specific antigen (PSA) levels. 2 Prostate cancer usually metastasizes to the regional lymph nodes such as pelvic lymph nodes (external and internal iliac); however, the involvement of the inferior gluteal lymph nodal group (part of internal iliac group) is quite uncommon. We herein report an unusual and unique case of prostate adenocarcinoma with involvement of bilateral inferior gluteal lymph nodes.

## Case Report

**Fig. 1:**
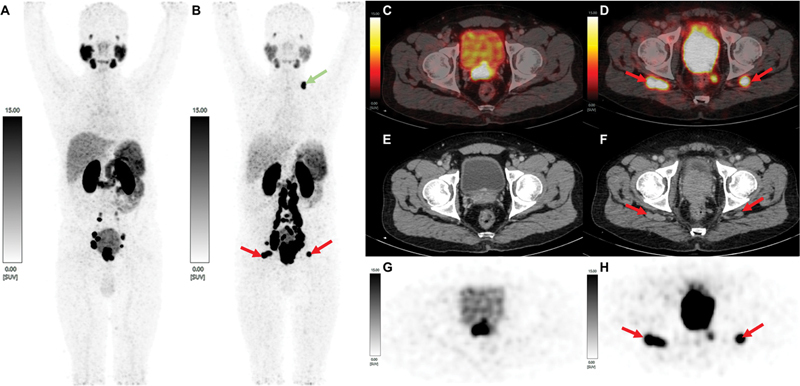
Maximum intensity projection of ^68^ Ga-PSMA PET scan at baseline ( **A** ) and after follow-up after 3 years postdefaulting ( **B** ), showing new onset bilateral inferior gluteal lymph nodes and left supraclavicular lymph nodes; baseline-fused PSMA PET/CT ( **D** ), CT ( **E** ), and PET ( **G** ) axial images showing tracer avid prostatic primary and no inferior gluteal lymphadenopathy. Follow-up-fused PSMA PET/CT ( **D** ), CT ( **F** ), and PET ( **H** ) axial images showing progression of local involvement of prostatic primary with new onset meso-rectal nodes and bilateral inferior gluteal lymphadenopathy.

**Fig. 2:**
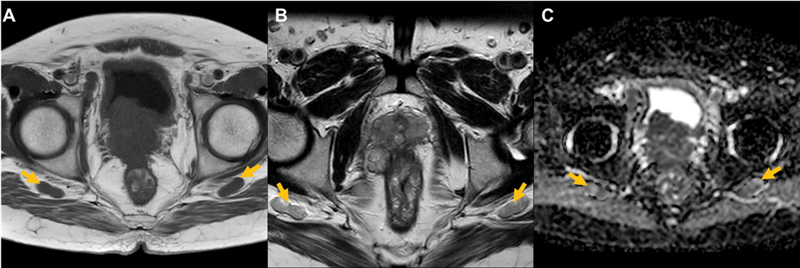
Axial T1 MRI ( **A** ), axial T2 MRI ( **B** ) and axial diffusion-weighted imaging MRI sequence ( **C** ) of inferior gluteal lymph node ( *marked in yellow* ).

A 42-year-old male with a medical history of tobacco chewing for 2 years and cigarette smoking in the past 23 years, initially presented with lower urinary tract symptoms (obstruction of urine flow). The patient had a Eastern Cooperative Oncological Group (ECOG) performance status of 1. The computed tomography (CT) urography showed a 2.6 × 2.6 × 3.2 cm ill-defined lesion in the prostate with loss of fat planes with the base of the urinary bladder and few enlarged internal and external iliac lymph nodes. Serum PSA levels at baseline were 40 ng/mL. A TRUS-guided biopsy of the prostatic lesion showed conventional prostatic adenocarcinoma with a Gleason Score of 5 + 4 = 9, WHO Grade Group 5 with no lympho-vascular invasion and no perineural invasion. Bone scan showed no evidence of bone metastasis. Baseline ^68^ gallium-prostate specific membrane antigen positron emission tomography/computed tomography ( ^68^ Ga-PSMA-11 PET/CT) scan showed prostate-specific membrane antigen (PSMA) expressing prostate lesions with PSMA expressing bilateral external and internal iliac, common iliac, and para-aortic lymphadenopathy. The patient was put on medical androgen deprivation therapy and was planned for external beam radiotherapy. He received 12 cycles of injection leuprolide for 3 months and defaulted due to the coronavirus disease (COVID-19) pandemic. Follow-up evaluation at a later date showed a raised PSA level of 148.4 ng/mL and ^68^ Ga-PSMA-11 PET/CT new onset PSMA expressing metastatic left supraclavicular node and new PSMA expressing metastatic intermuscular deposits in the bilateral gluteal region ( Figs. 1 and 2 ). The patient then underwent bilateral scrotal orchidectomy and was started on tablet abiraterone acetate due to rising serum PSA levels.

## Discussion

A review of literature revealed only one study report which detected enlarged inferior gluteal lymph nodes in a single case of prostate cancer with the help of multi-parametric magnetic resonance imaging). 3 To the best of our knowledge, ours is the only report of involvement of bilateral inferior gluteal lymph nodes in a case of prostate carcinoma on ^68^ Ga-PSMA-11 PET-CT.

Prostate cancer is the sixth leading cause of cancer death among men worldwide and the fifth highest incidence rate among males in India in 2016. 4 5 In high-risk prostate cancer, ^68^ Ga-PSMA-11 PET/CT is known to be a useful method for the detection of metastases. 6 Associated with the rise of prostate cancer, use of PSMA-PET/CT has been indicated for staging in high-risk prostate cancer, response assessment after therapeutic interventions in correlation with serum PSA levels, and evaluating biochemical recurrence and prior to planning patients for ^177^ Lu-PSMA-617 therapy. 6 Prostate cancer follows a predominant hematogenous mode of spread to bones via prostatic venous plexus draining into the vertebral veins and lymphatic mode to loco-regional lymph nodes. 7 The process of metastasis can be outlined through five key stages: (1) penetration of the basement membrane and movement into nearby tissue; (2) entry into either the bloodstream or lymphatic system; (3) survival while circulating within these systems; (4) exit from the vessels into tissue; and (5) establishment and growth of metastatic growths in secondary locations. 8

The posterior lobe of the prostate is drained via three primary pathways: (1) a lateral pathway draining to the external iliac lymph nodes. These lymph vessels also drain the terminal portion of the ductus deferens and seminal glands; (2) a laterodorsal pathway, which drains into the internal iliac nodes, via a course following the prostatic artery, and (3) a dorsal pathway, which drains to the sacral lymph nodes as well as the promontories and common iliac lymph nodes. The lymphatics drained from the anterior lobe of the prostate can be traced via two routes: (1) the majority of lymph drained from the anterior surface proceeds to the external iliac lymph nodes, via the paravesical space and (2) some vessels from the anterior lobe leave the prostate from the posterior surface, draining into a group of nodes known as inferior gluteal lymph nodes, which are part of the internal iliac lymph nodes. While the internal, external iliac, and obturator lymph nodes are the most frequently involved in prostate carcinoma, metastases to presacral and common iliac lymph nodes are relatively uncommon. The inferior gluteal nodes are located along the inferior gluteal artery and drain the prostate and the upper part of the urethra.

Albeit it is common to encounter loco-regional nodal disease, it is rare to detect the involvement of inferior gluteal lymph nodes in a case of metastatic prostate carcinoma. The gluteal lymph nodes belong to the parietal section of the internal iliac nodes. They can be categorized into two subdivisions: superior and inferior gluteal lymph nodes. These nodes are positioned alongside the corresponding blood vessels. These lymph nodes collect lymph from the deeper regions of the pelvis, including the gluteal subfascial and visceral tissues. The collected lymph subsequently flows into the internal and common iliac nodes before progressing to the lateral caval lumbar nodes. 3

A therapeutic implication to this may be that these nodes likely fall beyond the range covered by radiation beams and are not easily reachable through standard surgical procedures. As a result, they could have an impact on the way patients are clinically treated and on their prognosis.

This patient also had simultaneous involvement of left supra-clavicular lymph node and was detected on ^68^ Ga-PSMA-11 PET/CT and is frequently related to disseminated disease. 6 A potential explanation for the involvement of left supraclavicular lymph nodes is their proximity to the point where the thoracic duct enters the left subclavian vein. This close positioning could facilitate the possibility of retrograde spread allowing cancer cells to move against the natural flow of lymphatic drainage and reach these nodes. 9

## Conclusion

Bilateral inferior gluteal nodal metastasis is a rare phenomenon that may have an important implication for treatment planning and prognosis. The preferred imaging method is either a pelvic MRI or ^68^ Ga-PSMA-11 PET/CT. Their presence may alter the therapeutic radiation field coverage in cases where external radiotherapy is advocated modality of choice and, thus, appropriate detection may help in the correct management of the disease.
